# Molecular characteristics and stable carbon isotope compositions of dicarboxylic acids and related compounds in wintertime aerosols of Northwest China

**DOI:** 10.1038/s41598-022-15222-6

**Published:** 2022-07-04

**Authors:** Weining Qi, Gehui Wang, Wenting Dai, Suixin Liu, Ting Zhang, Can Wu, Jin Li, Minxia Shen, Xiao Guo, Jingjing Meng, Jianjun Li

**Affiliations:** 1 State Key Laboratory of Loess and Quaternary Geology, Key Lab of Aerosol Chemistry and Physics, Institute of Earth Environment, Chinese Academy of Sciences, Xi’an, 710061 China; 2 Key Laboratory of Geographic Information Science of the Ministry of Education, School of Geographic Sciences, East China Normal University, Shanghai, 200241 China; 3 School of Geography and the Environment, Liaocheng University, Liaocheng, 252000 China; 4 CAS Center for Excellence in Quaternary Science and Global Change, Xi’an, 710061 China; 5 National Observation and Research Station of Regional Ecological Environment Change and Comprehensive Management in the Guanzhong Plain, Xi’an, Shaanxi China

**Keywords:** Climate sciences, Environmental sciences

## Abstract

Dicarboxylic acids are one of the important water-soluble organic compounds in atmospheric aerosols, causing adverse effects to both climate and human health. More attention has therefore been paid to organic acids in aerosols. In this study, the molecular distribution and diurnal variations of wintertime dicarboxylic acids in a rural site of Guanzhong Plain, Northwest China, were explored. Oxalic acid (C_2_, day: 438.9 ± 346.8 ng m^−3^, night: 398.8 ± 392.3 ng m^−3^) is the most abundant compound followed by methylglyoxal (mGly, day: 207.8 ± 281.1 ng m^−3^, night: 222.9 ± 231.0 ng m^−3^) and azelaic (C_9_, day: 212.8 ± 269.1 ng m^−3^, night: 211.4 ± 136.7 ng m^−3^) acid. The ratios of C_9_/C_6_ and C_9_/Ph indicating that atmospheric dicarboxylic acids in winter in the region mainly come from biomass burning. Furthermore, secondary inorganic ions (NO_3_^−^, SO_4_^2−^, and NH_4_^+^), relative humidity, liquid water content, and *in-situ* pH of aerosols are highly linearly correlated with C_2_, suggesting that liquid phase oxidation is an important pathway for the formation of dicarboxylic acids. The δ^13^C analysis of C_2_ suggested that lighter carbon isotope compositions tend to be oxidized to form aqueous-phase secondary organic aerosols (aqSOA), leading to the decay of ^13^C in aqSOA products rather than aerosol aging. This study provides a theoretical basis for the mechanism of formation of dicarboxylic acid.

## Introduction

Secondary organic aerosols (SOAs), generated by physical and chemical transformation of volatile organic compounds (VOCs) as gaseous precursors in the atmosphere^1,2^, are closely related to some natural phenomena such as solar radiation, particle growth, cloud condensation nuclei formation, and reduced visibility^3,4^. Dicarboxylic acids, ketocarboxylic acids and α-dicarbonyls are important classes of SOAs, which mainly produced from photochemical oxidation with ozone or hydroxyl radicals (•OH)^5^ and aqueous processing^6^. Dicarboxylic acids and related polar organic compounds, water-soluble organic carbons (WSOC), can be always detected in urban^7^, mountain^8^, and remote region^9^. In addition, dicarboxylic acid is generally considered as an indicator of the degree of oxidation of WSOC in the atmosphere^10–12^, so it is quite necessary to explore the composition, source and oxidation mechanism of dicarboxylic acid in the atmosphere.

Domestic and foreign studies on the composition and source of dicarboxylic acid are mainly based more on the seasonal variations and trends therein in urban atmospheric aerosol^13,14^ but the diurnal variation of dicarboxylic acid in the rural of Northwest China is rarely studied. Guanzhong Plain, located in Northwest China, is one of the most heavily polluted regions in China with an annual average PM_2.5_ of more than 80 μg m^−3^^15^. It was reported that high levels of PM_2.5_ have a significant damaging effect on atmospheric visibility and human health^16^ in which water-soluble SOAs may play the vital roles since it not only scatters or absorbs visible light but amplifies the pollution solubility in the respiratory tract^17^. It is important to understand the source of water-soluble organic compounds. Stable carbon isotope analysis can provide available information about the sources and composition and formation mechanism of organic aerosols^18^. Therefore, isotope analysis, widely used, focuses on urban areas, such as Beijing^18,19^ and Xi’an to explore the source of atmospheric organic aerosols^20^. However, isotope analysis is rarely used to analyze the source and distribution of dicarboxylic acids in rural areas.

In this study, we measured dicarboxylic acids, ketocarboxylic acids, and α-dicarbonyls which collected in the rural area of Guanzhong Plain to study their sources and mechanisms of formation. The objectives of the present study are: (1) identify the source and composition of dicarboxylic acid in PM_2.5_ of atmospheric organic aerosol in the rural area of Northwest China; (2) assess the diurnal variations of these dicarboxylic acids and (3) reveal the mechanism of formation of dicarboxylic acid and related SOAs.

## Sampling and analysis

### Sample collection

In Lincun (34° 44′ N and 109° 32′ E, 354 m a.s.l.), a small village, is distancing 40 km northeast of Xi’an, the capital of Shaanxi Province, China. We collected PM_2.5_ samples from 20 January to 1 February during the winter in 2017. The aerosol sampler was located in the rooftop of a three-storey building. PM_2.5_ samples were collected by a mid-volume air sampler (Laoshan Company, China) at an airflow rate of 100 L min^−1^ on a pre-baked (450 °C for 12 h) quartz fiber filter (*Φ* 90 mm) on a day (08:00–19:00)/night (20:00–07:00) basis for 10 h each half-cycle. At the beginning and the end of the sampling, field blank samples were collected by mounting the blank filter onto the sampler for 10 min without sucking air. The total samples were sealed within an aluminum foil bag and stored at − 18 °C before the laboratory analysis^21^.

### Sample analysis

#### Dicarboxylic acids and related organic compounds

Dicarboxylic acids, ketocarboxylic acids, and α-dicarbonyls were analyzed and the methods can be found anywhere^22,23^. In short, firstly, Milli-Q water extracted ultrasonically a quarter of the filtrate three times. Then, under vacuum condition, the sample was concentrated to dryness by rotary evaporation and reacted with 14% BF_3_/butanol mixture at 100 °C for 1 h. Thereafter, the sample was derivatized and added to n-hexane, extracted by pure water three times. Finally, the hexane layer was concentrated to 100 μL by nitrogen blowing and quantified by gas chromatography (GC) (Agilent, GC7890A) coupled with an FID detector (DB-5MS, 30 m × 1.25 mm × 0.25 m).

The temperatures of GC oven was programmed to ascend from 50 to 120 °C at a gradient of 30 °C min^−1^, and then at a rate of 6 °C min^−1^ to 300 °C and hold at 300 °C for 10 min. The target compounds recoveries of oxalic acid could reach to 80–85% and 92–115% for others. In the field blanks, target compounds were less than 5% of those in actual specimens. Data reported here were corrected by use of field blank specimens but not by the recoveries.

#### Stable carbon isotope composition of dicarboxylic acids and related SOAs

The shorter chain dicarboxylic acids and related SOAs could be measured by the stable carbon isotopic compositions (δ^13^C), described by Aggarwal and Kawamura^22^. Briefly, the samples δ^13^C values were detected by gas chromatography–isotope ratio mass spectrometry (GC-IR-MS; Thermo Fisher, Delta V Advantage). Then, based on the measured δ^13^C values of derivatives and the derivatizing agent (BF_3_ = n-butanol) we used isotopic mass balance equation to calculate the δ^13^C values of free organic acids^24^. We measured three times for every sample to ensure the analytical error of the δ^13^C values less than 0.2‰. The δ^13^C data reported here are averaged values of the triplicate measurements.

#### Carbonaceous fractions and water-soluble inorganic ions

Elemental carbon (EC) and organic carbon (OC) were applied by a DRI Model 2001 Carbon Analyzer following the Interagency Monitoring of Protected Visual Environments (IMPROVE) Thermal/Optical Reflectance (TOR) protocol performed by Yang^25^. Inorganic ions and water-soluble organic carbons was extracted by the conventional method as detailed elsewhere^26^. Besides, the liquid water content (LWC) and *in-situ* (pH_is_) of particles were calculated by ISORROPIA-II^27^.

## Results and discussion

### Overview of meteorological conditions and chemical components

**Figure 1:**
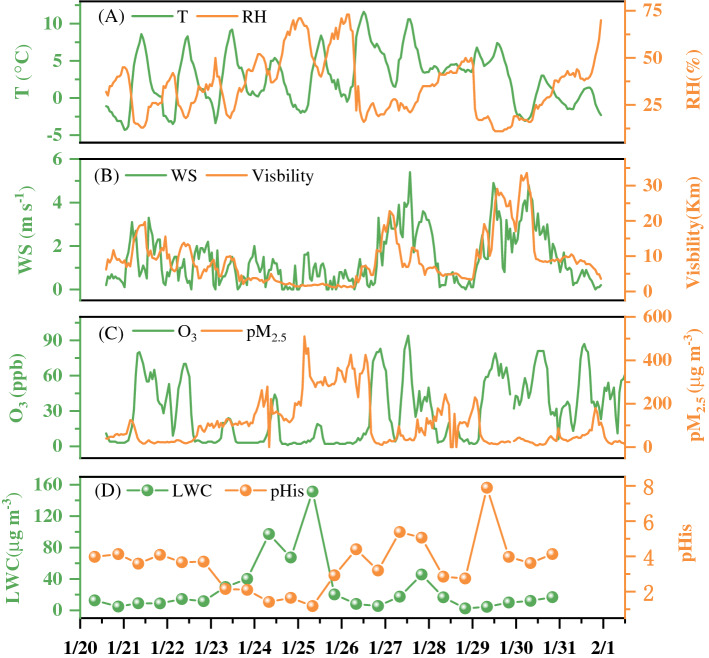
Diurnal variations of temperature (T), relative humidity (RH), wind speed (WS), visibility concentrations of O_3_, PM_2.5_, liquid water content (LWC) and pHis.

Diurnal variations of meteorological parameters and the determined dicarboxylic acids (α-dicarbonyls, keto-carboxylic acids and dicarboxylic acids) are illustrated in Fig. 1. During the sampling periods, relative humidity (RH), PM_2.5_ concentrations and LWC reach their peak (Fig. 1A,C,D) while wind speeds (WSs) (Fig. 1B) are lowest. As shown in Table 1, the mass concentration of PM_2.5_ ranged from 54.0 to 430.6 µg m^−3^, with mean values of 189.8 ± 91.7 and 181.1 ± 107.6 μg m^−3^ in day-time and night-time, respectively. It was much higher than the national air quality standard of 75 μg m^−3^, indicating serious air pollution in the rural region of Northwest China. OC and EC were 44.0 ± 17.9 and 13.6 ± 6.8 μg m^−3^ by day and 47.9 ± 28.4 and 14.1 ± 5.3 μg m^−3^ by night, respectively. Our results were lower than those measured in Xi’an (OC: 59 ± 28, EC: 19 ± 8.2)^28^. The OC/EC was 3.1 ± 0.3 μg m^−3^ by day and 3.4 ± 0.4 μg m^−3^ by night, indicating almost no difference between day and night. The LWC values were 21.2 ± 20.83 (day) and 33.86 ± 46.74 μg m^−3^ (night), which were significantly lower than those of LWC (94 ± 100 μg m^−3^ by day and 75 ± 69 μg m^−3^ by night) at top Mount Tai, which was possibly due to the high RH at its summit. pH_is_ values were 3.43 ± 3.36 and 3.65 ± 1.89 μg m^−3^ by day and night, respectively.

**Table 1:** Concentrations of dicarboxylic acids, keto-carboxylic acids, α-dicarbonyls, OC, and EC in PM_2.5_ of Weinan, China by day and night.

	Day (n = 12)	Night (n = 12)
*I. Dicarboxylic acids, ng m*^*−3*^		
Oxalic, C_2_	438.9 ± 346.8	398.8 ± 392.3
Malonic, C_3_	39.5 ± 36.6	35.8 ± 35.3
Succinic, C_4_	57.9 ± 33.4	59.6 ± 47.1
Glutaric, C_5_	15.8 ± 10.4	16.6 ± 13.6
Adipic, C_6_	10.0 ± 4.1	9.7 ± 5.8
Pimelic, C_7_	10.9 ± 5.6	10.2 ± 7.7
Suberic, C_8_	7.2 ± 4.3	7.5 ± 5.9
Azelaic, C_9_	212.8 ± 269.1	211.4 ± 136.7
Undecanedioic, C_10_	38.2 ± 43.2	30.4 ± 26.8
Undecanedioic, C_11_	6.7 ± 3.8	6.6 ± 5.3
Mehtylsuccinic, iC_5_	9.5 ± 6.0	9.1 ± 7.4
Methylglutaric, iC_6_	7.9 ± 10.3	8.0 ± 10.6
Maleic, M	6.7 ± 11.0	9.3 ± 16.1
Fumaric, F	8.9 ± 7.3	8.5 ± 7.2
Phthalic, Ph	68.2 ± 28.8	63.6 ± 33.1
Isophthalic, iPh	5.8 ± 3.9	6.4 ± 6.3
Terephthalic, tPh	3.2 ± 2.4	4.6 ± 3.7
Subtotal	940.3 ± 967.8	903.8 ± 727.1
*II. Keto-carboxylic acids, ng m*^*−3*^		
Pyruvic, Pyr	15.4 ± 6.6	14.9 ± 4.9
Glyoxylic, ωC_2_	44.9 ± 31.2	37.1 ± 30.6
7-Oxoheptanoic, ωC_7_	8.3 ± 4.6	7.5 ± 4.5
Ketoacids	60.3 ± 35.1	52.0 ± 34.5
Subtotal	128.8 ± 73.7	111.6 ± 72.6
*III. α-Dicarbonyls, ng m*^*−3*^		
Glyoxal, Gly	21.7 ± 15.3	19.4 ± 15.1
Methylglyoxal, mGly	207.8 ± 281.1	222.9 ± 231.0
Subtotal	229.5 ± 294.1	223.7 ± 243.5
*IV. Others, μg m*^*−3*^		
OC	44.0 ± 17.9	47.9 ± 28.4
EC	13.6 ± 6.8	14.1 ± 5.3
PM_2.5_	189.8 ± 91.7	181.1 ± 107.6
LWC	21.2 ± 20.83	33.86 ± 46.74
pHis	3.43 ± 3.36	3.65 ± 1.89
*V. Carbon isotope*		
δ^13^C	− 20.50 ± 3.30	− 19.94 ± 3.61

**Figure 2:**
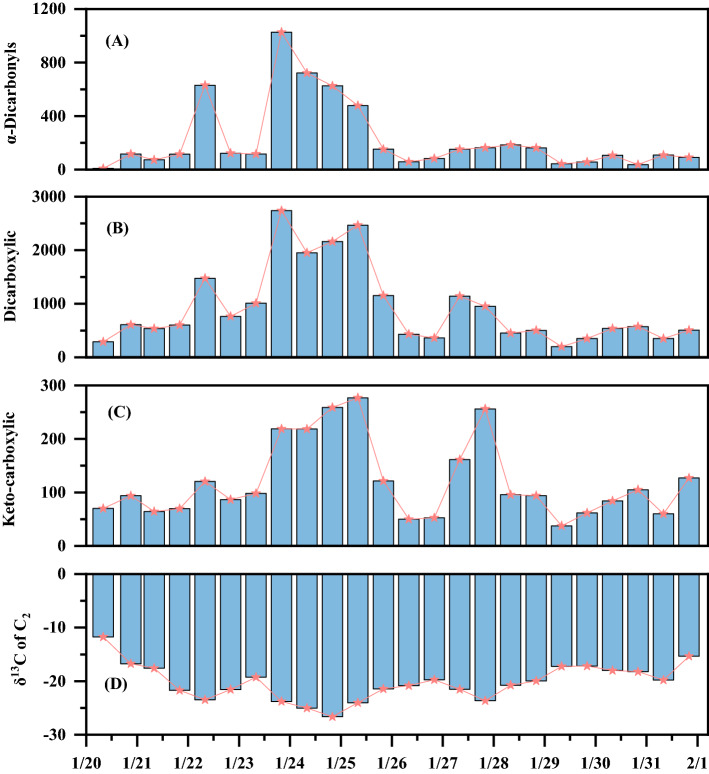
Diurnal variations of α-dicarbonyls, keto-carboxylic acids, and dicarboxylic acids.

Concentrations of total dicarboxylic acids (940.3 ± 967.8 ng m^−3^), keto-carboxylic acids (128.8 ± 73.7 ng m^−3^), and α-dicarbonyls (229.5 ± 294.1 ng m^−3^) by day were higher than those (903.8 ± 727.1, 111.6 ± 72.6, and 223.7 ± 243.5 ng m^−3^) by night. The concentrations of total dicarboxylic acids and related SOAs fluctuated significantly, with a maximum (2161 ng m^−3^) on 24 and 25 Jan, 2017 and minimum (37.6 ng m^−3^) on 31 Jan. (Fig. 2B). Dicarboxylic acid increased with the increase of LWC, suggesting enhanced formation of dicarboxylic acid through liquid oxidation in (Fig. 1D), while high pH is not conducive to the formation of dicarboxylic acid. This result was consistent with previous research results^29,30^. α-dicarbonyls and keto-carboxylic also exhibited the same fluctuating trend as dicarboxylic acid in Fig. 2A and C. We found that high RH and low WS are beneficial to the transformation and generation of Dicarboxylic acid. The minimum WS is not conducive to the migration and diffusion of dicarboxylic acids, leading to the accumulation of SOAs^31^. The results indicated that atmospheric meteorological parameters are closely related to the change of dicarboxylic acids in the environment, and our experimental results verify the previous research conclusions^32^.

On 28 Jan. (the traditional Chinese Spring Festival), the concentration of dicarboxylic acid was significantly reduced. Meanwhile, the concentrations of K^+^, Ca^2+^, and Mg^2+^ were 22.53, 2.81, and 0.74 µg m^−3^, respectively in Table 2, reaching their maxima^31^. Metal ions such as K^+^, Ca^2+^, and Mg^2+^ were common in fireworks^33^. During the festival, local residents set off fireworks containing metal ions, which could be released into the atmosphere and react with the dicarboxylic acids to form a stable complex (such as calcium oxalate). Therefore, the content of dicarboxylic acid decreased significantly during Spring Festival. Our results are similar to those reported in previous studies^34^.

**Table 2:** Concentrations of K^+^, Ca^2+^, and Mg^2+^.

Ions	K^+^	Ca^2+^	Mg^2+^
Concentration (µg m^−3^)	22.53	2.81	0.74

### The molecular composition of dicarboxylic acids and its related compounds

**Figure 3:**
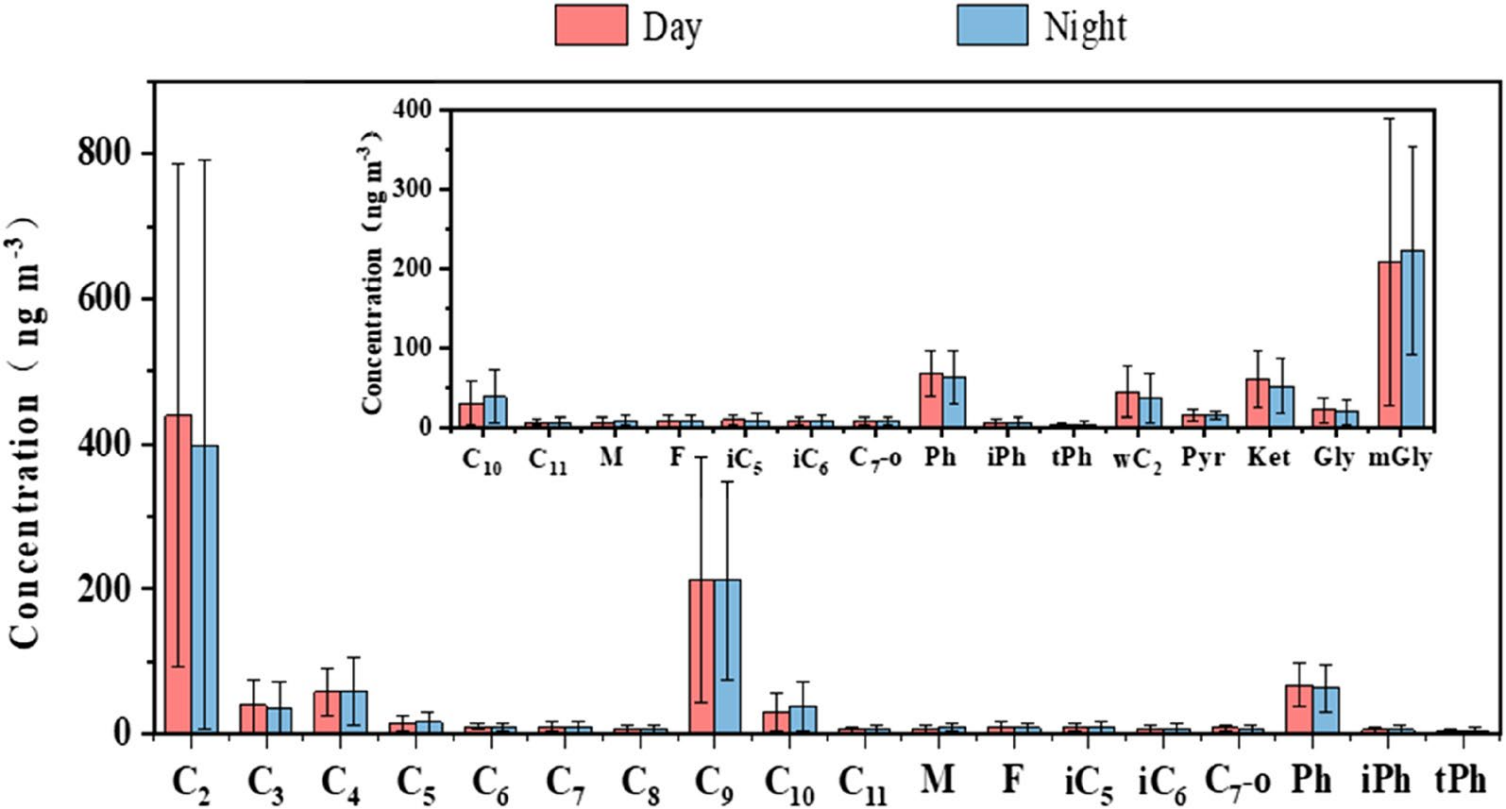
Molecular compositions of dicarboxylic acids and related compounds in PM_2.5_ over Weinan.

The molecular composition of dicarboxylic acids and related compounds is demonstrated in Fig. 3. C_2_, (day: 438.9 ± 346.8 ng m^−3^, night 398.8 ± 392.3 ng m^−3^) is the most abundant dicarboxylic acid, followed by methylglyoxal (mGly), azelaic acid, and phthalic acid (Ph, day: 68.2 ± 28.8 ng m^−3^, night: 63.6 ± 33.1 ng m^−3^) in dicarboxylic acids. The concentrations of C_9_ are 212.8 ± 269.1 ng m^−3^ and 211.4 ± 136.7 ng m^−3^ by day and by night. In addition, mGly contents by day (207.8 ± 281.1 ng m^−3^) are equal to those at night (222.9 ± 231.0 ng m^−3^), accounting for more than 90% of the total α-dicarbonyls. Gly concentrations at 21.7 ± 15.3 ng m^−3^ by day and 19.4 ± 15 ng m^−3^ by night are significantly lower than those of mGly. The reason for this may be that, compared with Gly, mGly has stronger biogenic sources and the lower oxidation rate of with OH radicals in the aerosol phase^35,36^. These α-dicarbonyls may serve as precursors to SOAs through heterogeneous reactions^37^. Ketoacids (day: 60.3 ± 35.1 ng m^−3^, night: 52.0 ± 34.5 ng m^−3^) are dominant in the keto-carboxylic acids. Ranked second is glyoxylic acid (ωC_2_) at 44.9 ± 31.2 ng m^−3^ by day and 37.1 ± 30.6 ng m^−3^, by night, respectively. It was reported that ωC_2_ is originally formed from glyoxal photochemically oxidized with OH radicals and other oxidants in the aqueous phase, followed by further oxidation to oxalic acid^21,38^.

### Possible sources and mechanism of formations of dicarboxylic acids and related compounds

**Figure 4:**
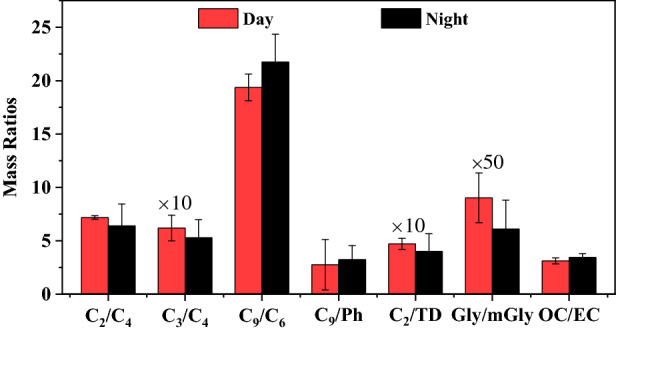
Diurnal variations of mass ratios of C_2_/C_4_, C_3_/C_4_, C_9_/C_6_, C_9_/Ph, C_2_/TD, Gly/mGly, and OC/EC (TD: total dicarboxylic acids; the mass ratios of C_2_/TD, C_3_/C_4_, and Gly/mGly expanding 10 and 50 times, respectively).

The characteristic ratios can be used to investigate the sources and formation process of dicarboxylic acids and related compounds^39^. Ratios of, C_2_/C_4_, C_3_/C_4,_ C_9_/C_6_, C_9_/Ph, C_2_/TD, Gly/mGly, and OC/EC are displayed in Fig. 4. C_2_ is formed by photochemical oxidation of various precursors with hydroxyl radicals (·OH)^40^. C_4_ is hydroxylated to form hydroxy succinic acid (*h*C_4_), which can further form C_2_ and C_3_^14^. The ratio of C_3_/C_4_ is useful for evaluation of photochemical oxidation degree since C_3_ is produced from C_4_ by photochemical oxidation. Thus, the ratios of C_2_/C_4_ and C_3_/C_4_ are used to characterize the photochemical oxidation of organic aerosols^41^. The ratio of C_2_/C_4_ is 7.8 ± 1.70 by day and 6.4 ± 0.35 by night, respectively, similar to those in a mountainous atmosphere (8.0 ± 2.7)^42^, lower than those in the North and South Pacific (8.7)^43^, and higher than that from vehicle exhausts (4.1)^21^. The C_3_/C_4_ ratio does not differ much between day (0.62 ± 0.22) and night (0.53 ± 0.27), but was significantly lower than on the Tibetan Plateau (2.2 ± 1.3) in summer^35^. A strong correlation between C_3_/C_4_ and ambient temperature has been reported, proving that high temperatures contribute to the photochemistry of organic aerosols^44^. However, such correlation is not found in this study. C_2_/TD was similar by day (0.47 ± 0.05) and night (0.4 ± 0.16). These results suggest that photochemical aging is insignificant.

Previous study found that C_6_ and Ph are mainly produced by the oxidation of anthropogenic cyclohexene and aromatic hydrocarbons^45,46^. On the contrary, C_9_ is mainly produced by the oxidation of biogenic unsaturated oleic acid, which contains a double bond at the C-9 position^47^. Thus, both ratios of C_9_/C_6_ and C_9_/Ph are indicative of the source strengths of biogenic *versus* anthropogenic emissions. The ratio of C_9_/C_6_ is higher by night (21.75 ± 5.61) than by day (19.37 ± 20.25), which is related to the higher emissions from biomass burning sources in rural areas by night. The ratios of C_9_/Ph are 2.75 ± 2.36 (day), and 3.23 ± 1.32 (night), respectively. Our results are lower than those measured at top Mount Tai (7.2 ± 2.2) in summer^21^. It is more likely that the photochemical reaction of unsaturated fatty acids emitted by biological sources on Mount Tai in summer is more intense than that in the rural regions investigated during this study in winter.

The ratios of Gly/mGly produced by anthropogenic and biological sources are 1:1 and 1:5, respectively^48^. Since the absorption coefficients of Gly and mGly are similar, when the biological source is a single emission source, the ratio of Gly/mGly should theoretically be 1:5. In contrast, when the anthropogenic source is a single emission source, Gly/mGly should be 1:1. In this study, the Gly/mGly ratio is 0.18 ± 0.15 by day and 0.12 ± 0.05 by night, which was close to 1:5. However, as discussed above, as well as previous studies conducted in Guanzhong Plain, anthropogenic emission from biomass and/or fossil fuel combustion was considered as the predominant source of atmospheric aerosols in winter in the region. These results may suggest that mGly have another source or formation processes other than biological emission, and the exact explanation needs further research.

**Figure 5:**
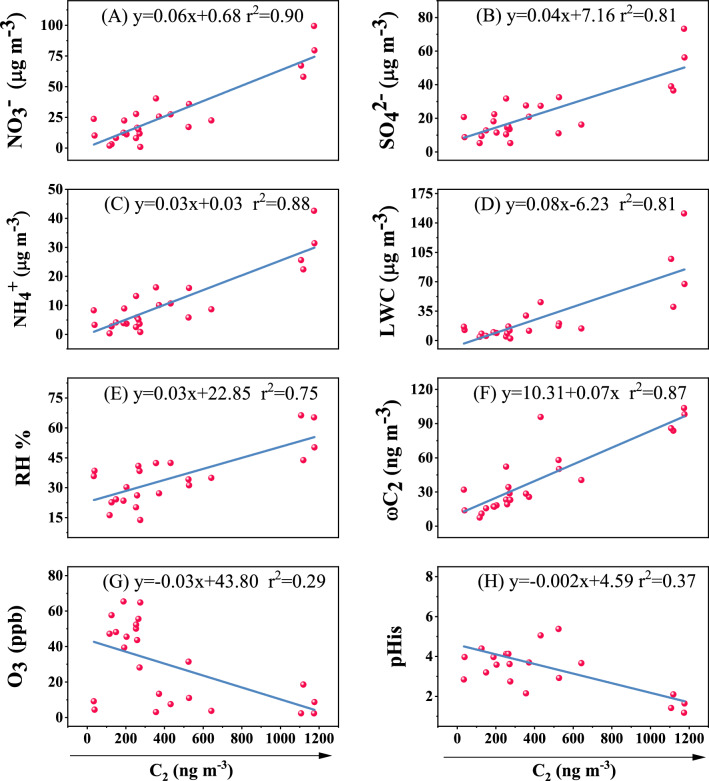
Linear regressions of oxalic acid (C_2_) with NO_3_^−^, SO_4_^2−^, NH_4_^+^, liquid water content (LWC), relative humidity (RH), O_3_, pH_is_, and ωC_2_.

The linear relationships between C_2_ and inorganic ions (NO_3_^−^, SO_4_^2−^, and NH_4_^+^), meteorological parameters (LWC, RH, O_3_, and pH_is_) and ωC_2_, are shown in Fig. 5. NO_3_^−^, SO_4_^2−^, and NH_4_^+^ play a critical role in the formation of dicarboxylic acid^49^. The linear correlation coefficient between NO_3_^−^ and C_2_ is 0.90, indicating a positive correlation between them (Fig. 5A). Our results are in accord with those of a previous study^50^. In Fig. 5B, SO_4_^2−^ also shows a similar effect, and the correlation coefficient between SO_4_^2−^ and C_2_ is 0.81. The sulphates (SO_4_^2−^) in the atmosphere mainly come from the liquid-phase oxidation of SO_2_ emitted from coal burning on the surface of fine particles^51^, as favored by high-temperature, humid conditions^52^. The results confirm that C_2_ is mainly formed by liquid oxidation. In addition, NH_4_^+^ also has a high linear correlation in Fig. 5C, with *r*^2^ reaching 0.88. This indicates that alkaline conditions are more conducive to the formation of C_2_. Consistent with the reaction results, alkalinity gradually increased with the decrease of acidity of atmospheric particles, contributing to the transformation of dicarboxylic acid precursor to C_2_^53^. NO_*x*_ will form NO_3_^−^ in the liquid film through heterogeneous reaction, and then absorb NH_4_^+^ in the air. Nitrate on the surface of particulate matter will further enhance the water absorption of atmospheric particles, expand the liquid phase on the surface, and provide a possibility for the dissolution and reaction of SOAs^54^. Therefore, our earlier studies proved that dicarboxylic acids are mainly derived through SOA formation via reactions in aqueous phase^55,56^.

In Fig. 5D, the *r*^2^ value of LWC is 0.81, indicating that C_2_ is mainly produced by the distribution of precursor from gas to liquid phase and the subsequent liquid phase process. The increase in LWC contributes to the generation of SOAs^57^. In addition, the LWC of atmospheric aerosol is mainly affected by RH and inorganic salt ions^58^. C_2_ is positively correlated with RH, and its linear correlation coefficient is 0.75 (Fig. 5E). Previous studies show that RH and LWC have a linear relationship with the formation of C_2_, indicating that a wet environment is conducive to the formation of a C_2_ aqueous phase^30,59^. Our experimental results confirm the previous research again that the binary carboxylic acids in the atmosphere mainly come from liquid phase oxidation^6^. Glyoxylic acid (ωC_2_) is an intermediate product of the liquid phase reaction of dicarboxylic acid. C_2_ and ωC_2_ have a good linear correlation, as illustrated in Fig. 5F, with a linear correlation coefficient of 0.87. The concentration of C_2_ increases with the increase of the concentration of ωC_2_, again proving that oxalic acid is formed by liquid-phase oxidation.

There is a significant negative correlation between C_2_ and O_3_ and pH_is_, and the linear correlation coefficients are only 0.29 and 0.37 in Fig. 5G and H, respectively. This reveals that the roles of O_3_ and pH_is_ are not obvious in this research, however, as reported, O_3_ is an oxidizing substance in the atmosphere, and increasing O_3_ concentration is conducive to the transformation of primary pollutants into secondary pollutants^60^. It may be that dicarboxylic acids and their compounds are transformed through liquid-phase oxidation, which may be related to other oxidants, such as H_2_O_2_, HO radical and NO_3_ radical. The acidity of organic aerosols was found to be related to the formation of biological SOAs^61^. C_2_ is negatively correlated with pH_is_, which is possibly because strongly acidic conditions can inhibit the generation of dicarboxylic acid and its precursors. Furthermore, Increasing RH can reduce the relative acidity, which is not conducive to the formation of dicarboxylic acid in acid catalytic reactions^62^.

### Diurnal variations in stable carbon isotopes of major dicarboxylic acids and related compounds

The further to understand the mechanism of formation of C_2_, the δ^13^C value of organic dicarboxylic acid by using the isotope mass balance equation for analyzing the degree of aging of aerosols. The diurnal variations of stable carbon isotopes of C_2_ are illustrated in Fig. 2D, with the day and night-time ranges of − 20.50 ± 3.30 (− 15.33 to − 26.64‰) and − 19.94 ± 3.61 (− 11.73 to − 25.03‰), respectively. The δ^13^C of C_2_ values of oxalic acid in Sapporo aerosol is the highest (− 14.0 to − 22.4‰, mean: − 18.8‰)^22^ and ranges from − 20.5 to − 10.1 at the Korea Climate Observatory at Gosan in winter^50^. Our results match those arising from previous studies. The trend in the variations of C_2_ isotope content is more negative in the first five days. The reason for this phenomenon is that in the process of SOAs generated by the oxidation of precursor VOC, carbon atoms with lighter isotopes in the gas phase tend to be oxidized and enter the aerosol phase in the form of reaction products, forming SOAs^63^. Therefore, SOA formation causes the attenuation of ^13^C in aerosols (δ^13^C reduction). At the same time, as shown in Fig. 1D, LWC also reached its maximum value. Due to the dynamic isotope fractionation effect, VOC and SVOC precursors with lighter carbon isotope compositions tended to be oxidized to form aqSOA, resulting in the decline of ^13^C in aqSOA products^64^. Thus, the liquid phase generation pathway of dicarboxylic acid in this study is VOC → SVOCs → Pyr → ωC_2_ → C_2_. Subsequently, δ^13^C gradually shows an upward trend on 28 Jan. This is probably because Chinese traditional festivals focus on setting off fireworks and firecrackers, releasing many pollutants including some reactive metals such as Ca and K. The emitted metals could react with dicarboxylic acid to form water-insoluble oxalates, which cannot be measured by the analyzing method in this study. This meant that the formation of secondary oxalic acid was relatively weaker, resulting its carbon isotopes rise slightly.

## Conclusion

In this study, total dicarboxylic acids, keto-carboxylic acids, α-dicarbonyls, EC, OC, PM_2.5_, LWC, and pH_is_ from 20 January to 1 February 2017 in a rural site on Guanzhong Plain, Northwest China were analyzed. The mass concentration of PM_2.5_ in the atmospheric aerosol ranged from 54.0 to 430.6 μg m^−3^, significantly exceeding the national standard. Total dicarboxylic acids and ketocarboxylic acids and α-dicarbonyls fluctuated regularly with LWC and RH. Molecular composition of dicarboxylic acids in the aerosol samples was characterized by the predominance of oxalic (C_2_) acid, methylglyoxal (mGly), and azelaic (C_9_) acid. The source of pollution from dicarboxylic acid was mainly biological as evinced by C_9_/C_6_, C_9_/Ph, and Gly/mGly. There was a significant correlation between C_2_ and LWC, which proved that C_2_ was mainly produced by the distribution of precursor from gas to liquid phase and the subsequent liquid-phase process. In addition, C_2_ concentration increased with the increase in the concentration of ωC_2_, the intermediate product of the liquid-phase reaction, which also confirmed the importance of the liquid-phase formation of oxalic acid. NO_3_^−^, SO_4_^2−^, and NH_4_^+^ played an important role in the formation of C_2_ and its related compounds. The C_2_ isotope data implied that SOAs were caused by precursor VOC oxidation rather than photochemical cracking of long-chain diacids.

## Data Availability

The datasets used and/or analysed during the current study available from the corresponding author on reasonable request.
